# Beyond Couch Correction in Head and Neck Radiotherapy: A Narrative Review of Action-Oriented Positioning Management

**DOI:** 10.3390/cancers18142249

**Published:** 2026-07-14

**Authors:** Kouta Hirotaki, Hajime Oyoshi, Kana Motegi, Atsushi Motegi, Masashi Ito, Sadamoto Zenda

**Affiliations:** 1 Department of Radiological Technology, National Cancer Center Hospital East, 6-5-1 Kashiwanoha, Kashiwa 277-8577, Japan; 2 Section of Radiation Safety and Quality Assurance, National Cancer Center Hospital East, 6-5-1 Kashiwanoha, Kashiwa 277-8577, Japan; 3 Department of Radiation Oncology, National Cancer Center Hospital East, 6-5-1 Kashiwanoha, Kashiwa 277-8577, Japan

**Keywords:** head and neck radiotherapy, positioning, immobilization, image-guided radiotherapy, adaptive radiotherapy, artificial intelligence

## Abstract

Accurate positioning remains a central challenge in head and neck radiotherapy because highly conformal dose distributions are delivered near multiple critical organs, and clinically relevant discrepancies may not be resolved by rigid image-guided correction alone. This narrative review synthesizes evidence on positioning management in head and neck radiotherapy, with particular emphasis on the transition from couch correction to action-oriented clinical decision-making. Peer-reviewed studies, guidelines, and key reviews published between January 2005 and April 2026 were identified using PubMed/MEDLINE and reference screening. Positioning uncertainty is often region-specific and dynamic, reflecting lower-neck and shoulder mismatch, mandibular variation, mask-fit deterioration, functional motion, and progressive anatomical changes during treatment. Thermoplastic immobilization, customized headrests, bite blocks, cone-beam computed tomography, six-degree-of-freedom correction, and adaptive workflows have improved reproducibility and verification; however, these approaches do not eliminate mismatches caused by non-rigid posture, immobilization failure, or evolving anatomy. In such situations, the key clinical task is to distinguish rigid setup deviations suitable for online correction from discrepancies requiring repositioning, re-immobilization, repeat simulation, or adaptive replanning. Emerging technologies, including artificial intelligence, dose-of-the-day estimation, corrected CBCT, synthetic CT, rapid replanning, and workflow-integrated decision support, may help close the gap between mismatch detection and intervention selection. Positioning management should therefore evolve from a correction-centered model to an action-oriented framework that links observed geometric or anatomical change to the most appropriate clinical response.

## Simple Summary

Head and neck radiotherapy requires highly reproducible patient positioning because treatment fields are close to critical organs such as the spinal cord, brainstem, salivary glands, and swallowing-related structures. Modern image guidance and six-degree couch correction can improve setup accuracy, but they cannot fully correct non-rigid posture differences, regional mismatch, mask-fit deterioration, or anatomical changes during treatment. This review explains why the main clinical challenge is no longer simply detecting mismatch, but deciding what action should follow. We summarize current evidence and future technologies that may help treatment teams determine when couch correction is sufficient and when repositioning, re-immobilization, repeat simulation, or adaptive replanning is needed.

## 1. Introduction

Accurate patient positioning is a central requirement in head and neck intensity-modulated radiotherapy (IMRT), in which highly conformal dose distributions are delivered near multiple critical organs, including the spinal cord, brainstem, salivary glands, and swallowing-related structures. Residual geometric uncertainty is usually managed using planning margins, with clinical target volumes commonly expanded by 3–5 mm to generate planning target volumes and serial organs, such as the spinal cord or brainstem, which are often assigned planning organ-at-risk volume margins of 2 mm. These margins indicate that in head and neck IMRT, millimeter-scale deviations are not negligible, particularly in regions with steep dose gradients. However, margin-based compensation cannot fully resolve the complex regional mismatches caused by lower neck and shoulder displacement, mandibular rotation, swallowing-related laryngeal motion, weight loss, or progressive anatomical changes during treatment. Therefore, even in the era of advanced immobilization, image guidance, six-degrees-of-freedom correction, and adaptive radiotherapy, positioning uncertainty remains a clinically relevant limitation that requires a systematic evaluation that goes beyond couch-based correction [1,2].

Over the past two decades, the management of positioning uncertainty in head and neck radiotherapy has evolved. Contemporary practice now incorporates improved immobilization approaches, volumetric image guidance, rotational correction with six-degree-of-freedom couches, adaptive radiotherapy workflows, and advanced delivery techniques, such as stereotactic treatment, MR-guided radiotherapy, and non-coplanar or trajectory-based delivery. Simultaneously, open-face immobilization, surface-guided workflows, and patient-specific oral positioning devices have been used to expand the concept of positioning beyond simple rigid fixation. These developments have undoubtedly improved precision; however, the underlying problem has not been eliminated. Rather, they have clarified that positioning uncertainty in the head and neck region is anatomically heterogeneous, dynamically evolving during treatment, and only partly addressable with rigid image-based correction alone [3,4].

This issue is important because head and neck radiotherapy is affected by lower neck and shoulder mismatch, mask fit deterioration, weight loss, tumor regression, and other treatment-related anatomical changes. Thus, the practical challenge now extends to determining when immobilization, repositioning or re-immobilization, and adaptive replanning are necessary. The field is moving from a correction-centered model toward a broader framework of positioning management [4,5,6,7,8,9,10].

This narrative review therefore reframes head and neck positioning management around an unresolved clinical question: how should treatment teams act when the observed mismatch cannot be adequately resolved by couch correction alone? We integrate evidence on sources of positioning uncertainty, immobilization, daily setup, image guidance, technology-specific positioning requirements, repositioning, re-immobilization, adaptive response, and emerging decision-support technologies. The objective is not only to summarize current practice, but also to organize the evidence around an action-oriented framework that links mismatch detection to clinically meaningful interventions.

## 2. Search Strategy and Selection Criteria, and Terminology

This narrative review was structured with reference to published quality-assessment principles for narrative review articles [11]. We searched PubMed/MEDLINE for peer-reviewed articles published from January 2005 to April 2026 using combinations of terms related to head and neck radiotherapy, positioning, setup error, immobilization, image-guided radiotherapy, cone-beam computed tomography, six-degree-of-freedom correction, adaptive radiotherapy, surface-guided radiotherapy, and artificial intelligence. We also screened reference lists of relevant guidelines, reviews, and original studies.

We selected articles that were clinically or technically relevant to positioning management in head and neck radiotherapy, particularly studies addressing regional mismatch, immobilization failure, image-guided correction, adaptive response, or emerging decision-support technologies. Articles were excluded when they were unrelated to head and neck radiotherapy, did not address positioning or image-guidance issues, or were outside the scope of the review. As this was a narrative review, no formal meta-analysis or quantitative risk-of-bias assessment was performed. For terminology, positioning uncertainty was used as a broad concept that includes measurable setup errors and anatomical mismatches arising from regional deformation, immobilization variation, functional motion, or progressive anatomical change. The term mismatch was used particularly for regional, non-rigid, or anatomically discordant findings that may not be fully resolved by couch correction.

## 3. Sources of Positioning Uncertainty

Positioning uncertainty is significant in head and neck IMRT because millimeter-scale geometric deviations may remain relevant even when conventional planning margins are applied. Dosimetric studies have shown the measurable effects of setup uncertainty on target coverage and normal tissue doses [1,2].

Importantly, the positioning uncertainty in the head and neck region cannot be understood solely as a rigid translational problem. Setup deviations vary across anatomical subregions, with postures involving the neck, especially the lower neck, being less reproducible than those involving the cranial regions. Giske et al. demonstrated that local deformations persisted even with dedicated immobilization and were not adequately represented by a single global registration. Using megavoltage cone-beam computed tomography (CBCT), Motegi et al., reported that most deviations were approximately ±8 mm in the neck region and ±5 mm in the head region. Djordjevic et al. further reported that the systematic and random components of subregional deviations were 0.6–2.3 mm and 1.0–1.7 mm, respectively, despite daily image guidance. These findings indicate that setup deviations differ by anatomical sub-region and should not be interpreted as whole-volume rigid displacements [7,12,13].

Clinically relevant uncertainties are associated with the lower neck and shoulder regions. Neubauer et al. reported an average interfractional shoulder motion of 2–6 mm and showed that superior shoulder displacement reduced lower neck target coverage [6]. Tachibana et al. further showed that shoulder deformations greater than 6 mm could affect volumetric modulated arc therapy (VMAT) dose distribution [9]. Region-dependent effects have also been reported in the lower neck and shoulders. Hirotaki et al. found that regional positioning deviations involving the vertebrae, clivus, and mandible were associated with dose-distribution deterioration and that mandibular rotation is correlated with parotid dose indices [10]. Thus, clinically meaningful mismatches can arise across multiple anatomical subregions [14,15].

Progressive anatomical changes that occur during treatment, such as weight loss, tumor shrinkage, nodal regression, and soft-tissue deformation are major sources of uncertainty, as they may alter both the immobilization fit and the geometric relationship between the targets and organs at risk. Adaptive radiotherapy reviews have identified such changes as the major reasons for replanning for head and neck cancer [4]. Contesini et al. suggested that positioning deterioration is multifactorial and may be influenced by treatment time, patient habitus, and immobilization-related factors [16].

The clinical relevance of setup error differs according to the treated subsite and target extent. For localized nasal cavity or sinonasal tumors, strict alignment of cranial bony structures is often required, whereas structures inferior to the mandible may be outside the treatment volume. In contrast, localized laryngeal radiotherapy is more affected by swallowing-related motion, and daily image guidance may require particular attention to the larynx and hyoid bone. For definitive treatment of oropharyngeal, hypopharyngeal, or nasopharyngeal cancers, elective nodal volumes are often extensive and are located close to multiple organs at risk, including the oral cavity, parotid glands, pharyngeal constrictor muscles, spinal cord, and brainstem. In these settings, reproducibility of neck curvature, mandibular position, head rotation, and shoulder position becomes clinically important. Nasopharyngeal cancer is particularly challenging because precise cranial alignment is required near the skull base and brainstem, while elective coverage may extend to the lower neck; therefore, cranially focused six-degree-of-freedom matching may leave clinically relevant residual mismatch in the lower neck.

The representative regional and functional mismatch patterns that may not be fully corrected by image-guided couch correction are summarized in Table 1. These data suggest that positioning uncertainty in head and neck IMRT is dynamic and anatomy-dependent rather than a simple rigid-body displacement. This review cannot define universal action-level thresholds for repositioning, re-immobilization, or adaptive replanning. Nevertheless, the values summarized in Table 1 may provide practical reference data for developing institution-specific action levels.

The table includes lower neck, shoulder, mandibular, and swallowing-related factors; measurement sites; and key quantitative findings. The studies differed in design, imaging methods, and endpoints; thus, the values should be interpreted as representative rather than directly comparable. Abbreviations: AP, anteroposterior; CC, craniocaudal; CTV, clinical target volume; IMPT, intensity-modulated proton therapy; LR, left–right; VMAT, volumetric modulated arc therapy.

## 4. Immobilization and Daily Treatment Setup

Immobilization is a key component of head and neck radiotherapy because a reproducible treatment geometry must be established before image guidance and couch-based correction are applied. Current guidance for radiation therapists places immobilization, patient positioning, and position verification within a unified workflow, emphasizing that an accurate daily treatment setup depends on the quality of simulation positioning and immobilization [3].

Although thermoplastic masks remain the standard for immobilization, mask fixation alone may not ensure adequate reproducibility across all anatomical regions. Compared with standard headrests, customized head and neck support improves setup performance, and cone-beam CT-based studies have suggested improved consistency in regions such as the chin, cervical spine, and shoulder line. These findings indicate that the headrest design is an important determinant of setup reproducibility [19,20,21]. Representative studies on immobilization approaches and the reported setup accuracies are summarized in Table 2.

Bite blocks and mouthpieces also contribute to reproducible head and neck positioning. In addition to separating target volumes from adjacent oral structures, these devices improve setup reproducibility by stabilizing mandibular, tongue, and oral cavity position [22,23,24].

Immobilization of the lower neck and shoulder region requires special attention, particularly because its reproducibility is not always linked to that of cranial alignment. Even when skull–base matching is acceptable, variations in shoulder position, neck posture, body habitus, and mask fit may produce a caudal–neck mismatch. Therefore, careful shoulder positioning, patient-specific support, and a consistent neck posture are important prerequisites for effective image-guided correction [6,9,16].

Open face masks and surface guided radiation therapy (SGRT)-assisted workflows may improve patient comfort and enable surface-based monitoring during setup and treatment. These approaches can maintain acceptable positional stability in selected patients; however, surface information cannot fully replace internal anatomical verification in detecting regional mismatches in head and neck radiotherapy [25,26,27,28].

**Table 2:** Representative studies of immobilization and setup accuracy in head and neck radiotherapy.

Study	n	Technique	Correction	Immobilization	Key Finding
Anderson et al., 2022 [21]	20	VMAT	6DoF + daily CBCT	Mask + standard/custom headrest	Custom headrest reduced translational setup errors versus standard headrest: 1.2–1.5 vs. 1.6–2.9 mm; rotational errors were 0.51–0.91° vs. 0.75–0.93°.
Rodrigues et al., 2019 [29]	30	VMAT	3/6DoF + daily CBCT	Mask + standard/custom headrest	Custom support + 6DoF reduced interfraction translation from 1.4 ± 0.7 to 0.8 ± 0.3 mm; intrafraction translation was 0.7–1.0 mm.
Wang et al., 2016 [30]	21	SRT	6DoF + ExacTrac	Mask + custom headrest + bite-block	Setup error (3D vector) decreased from 2.7 ± 1.4 to 0.4 ± 0.3 mm after correction; intrafraction motion was 0.9 ± 0.6 mm.
Kang et al., 2020 [31]	41	SRT	CyberKnife + Xsight	Mask + headrest ± vacuum bag	Intrafraction motion was 0.92 ± 0.58 mm without vacuum bag and 1.12 ± 0.88 mm with vacuum bag.
Mail et al., 2024 [19]	189	VMAT	6DoF + daily CBCT	Mask + standard/custom headrest + mouth bite	Translational setup errors: 1.21/1.00/1.07 mm vs. 0.83/1.05/0.85 mm (CC/AP/LR).
Malone et al., 2025 [27]	56	VMAT	3/6DoF + CBCT/SGRT	Closed- vs. open-face mask	Open-face masks showed comparable intrafraction stability to closed-face masks; CBCT mean translation/rotation deviations were <0.2 mm/<0.1°.
Essers et al., 2024 [28]	19	VMAT	6DoF + CBCT/SGRT	Maskless SGRT vs. mask	Intrafraction motion > 2 mm occurred in 19% of maskless fractions and 9% of masked fractions.

Studies were organized according to the number of patients, treatment technique, guidance or correction method, immobilization approach, and key quantitative findings. Mask refers to a thermoplastic immobilization mask, unless otherwise specified. The studies differed in design, imaging method, correction strategy, and endpoint; thus, the values should be interpreted as representative rather than directly comparable. Abbreviations: AP, anteroposterior; CBCT, cone-beam computed tomography; CC, craniocaudal; CTV, clinical target volume; IMRT, intensity-modulated radiotherapy; LR, left–right; SRT, stereotactic radiotherapy; SGRT, surface-guided radiotherapy; VMAT, volumetric modulated arc therapy; 3/6DoF, three- or six-degrees-of-freedom correction.

## 5. Positioning Requirements Across Treatment Technologies

Technological advances in head and neck radiotherapy have increased the importance of identifying geometric deviations that can be corrected onsite and those that require repositioning, re-immobilization, or adaptive replanning. In conventional fractionated IMRT and VMAT, highly conformal dose distributions and steep dose gradients make the treatment sensitive to both translational and rotational mismatches. Six-degree-of-freedom correction can improve setup reproducibility, particularly when combined with individualized head support; however, its effectiveness depends on reproducible simulation positioning and stable immobilization throughout the treatment [29,32].

This principle is more critical in stereotactic radiotherapy (SRT), where high dose per fraction and limited geometric tolerance leave little room for intrafraction motion. Although customized immobilization and bite-block-based approaches improve setup accuracy and motion control, the primary challenge lies in identifying when residual motion or postural shifts exceed the safely manageable range using image-guided correction alone [30,31,33].

Magnetic resonance-guided radiotherapy and adaptive workflows shift positioning management from image matching to daily anatomical evaluation. Improved soft tissue visualization may help detect tumor regression, weight loss, organ displacement, or mask-fit deterioration; however, adaptive intervention still requires a reproducible posture, compatible immobilization, and efficient workflow [34,35,36,37,38].

Non-coplanar and trajectory-based delivery techniques illustrate how advanced treatment delivery may require a setup-specific robustness evaluation. Although these approaches can improve organ-at-risk sparing by expanding the beam-angle freedom, their clinical value depends on whether such benefits are preserved under setup and delivery uncertainties [39]. Loebner et al. demonstrated that dynamic trajectory radiotherapy maintained lower average normal tissue complication probabilities for xerostomia and dysphagia than VMAT in most scenarios of uncertainty; however, the dose variation differed according to scenario and anatomical structure [40]. Similarly, Hirotaki et al. reported that in a robustness evaluation of biaxially rotational dynamic radiation therapy with robust optimization, parotid and oral cavity sparing was achieved, whereas some target-coverage metrics were more affected by setup uncertainty [41,42,43]. These findings suggest that non-coplanar delivery may require trajectory-specific consideration of setup accuracy, rotational mismatch, image guidance, and delivery reproducibility. Particle- and neutron-based modalities add another layer of sensitivity because setup errors may interact with range uncertainty, beam geometry, or biologically weighted dose distributions [44,45,46,47,48,49]. Particle therapy is particularly sensitive to setup error and anatomical change because it exploits characteristic physical properties, including the finite range and Bragg peak. In particle therapy, the therapeutic dose is concentrated near the end of the beam range; therefore, body contour change, tumor shrinkage, or setup error can have a greater dosimetric impact than in VMAT. Unlike the more robust dose distribution of VMAT, such changes in particle therapy may reduce target coverage. Therefore, strict positioning management is particularly important in head and neck particle therapy, where residual bony mismatch that cannot be fully corrected by six-degree-of-freedom matching may occur. BNCT also requires careful positioning management, because neutron flux decreases with distance from the irradiation aperture and patient setup can affect dose error in sitting-position head and neck treatments [48,49]. Therefore, across advanced delivery platforms, setup uncertainty should be evaluated not only as a geometric displacement but also as a treatment-specific factor that may affect target coverage and organ-at-risk sparing.

Among treatment technologies, some advanced modalities require more stringent positioning than conventional IMRT. In these settings, image guidance and six-degree-of-freedom correction are insufficient; clinicians must determine whether the observed bony or regional anatomical mismatch can be corrected by repositioning or whether it reflects a persistent anatomical change that should trigger adaptive replanning.

## 6. Image Guidance and Online Correction Strategies

Image guidance in head and neck radiotherapy has shifted from planar bony verification to volumetric imaging and structure-specific assessment. This suggests that external setup reproduction or a single global bony match alone does not ensure accurate treatment geometry [50,51,52]. CBCT has therefore become central to daily or protocol-based verification because it can identify regional mismatch and anatomical change that may not be adequately appreciated on planar images [52,53,54,55,56].

However, the clinical value of image guidance depends on the image interpretation. Whole-volume registration may improve overall alignment; however, residual mismatch may still be observed in clinically relevant subregions, such as the lower neck, shoulders, mandible, or laryngeal region [14,15,57,58]. Similarly, six-degree-of-freedom correction can reduce rigid translational and rotational errors but cannot fully resolve discrepancies caused by posture change, regional deformation, mask-fit deterioration, or progressive anatomical changes [29,32,51,59].

Thus, online image guidance should be used not only to calculate couch corrections but also to identify mismatches that may require repositioning, re-immobilization, or adaptive replanning. Automatic registration may improve workflow efficiency and consistency, but its reliability depends on the image quality, registration target, and the type of discrepancy being assessed [60]. Human oversight remains essential when image guidance is used to evaluate whether standard corrections suffice or if further intervention is required [50,51].

## 7. When Couch Correction Is Insufficient: Repositioning and Adaptive Response

**Figure 1:**
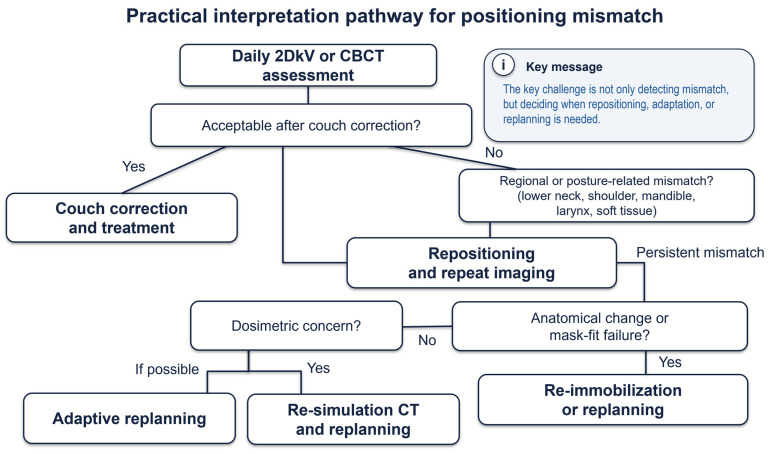
Practical interpretation pathway for positioning mismatch in head and neck radiotherapy. After daily 2DkV or CBCT assessment, discrepancies that are clinically acceptable after couch correction may proceed to couch correction and treatment. If residual mismatch remains unacceptable, regional or posture-related mismatch should prompt repositioning and repeat imaging. Persistent mismatch, anatomical change, or mask-fit failure should prompt further evaluation. Depending on dosimetric concern and clinical feasibility, subsequent actions may include adaptive replanning, re-simulation CT and replanning, or re-immobilization or replanning. This figure was created by the authors and was not reproduced or adapted from previously published material.

Image guidance and couch-based correction are essential in modern head and neck radiotherapy; however, they do not solve all clinically relevant positioning problems. In daily practice, the skull base or upper cervical spine may be well aligned after image matching, while residual mismatch remains in the lower neck, shoulders, mandible, or soft tissues. Such findings suggest a region-specific difference between the treatment and simulation geometries rather than a simple rigid setup error [7,13,14,15,57,61]. Figure 1 summarizes a practical interpretation pathway for escalating from couch correction to repositioning, re-immobilization, or replanning when positioning mismatch is not clinically acceptable after daily image guidance.

In such situations, additional couch shifts are not always appropriate. If the mismatch reflects neck flexion or extension, shoulder displacement, mandibular rotation, mask loosening, or poor patient posture, the first intervention should be repeat setup or repositioning. The aim is to restore the planned treatment posture before irradiation, rather than to optimize one anatomical region at the expense of another. This distinction is particularly important in head and neck IMRT, in which different anatomical levels may have different dosimetric priorities [6,7,9,10,13,61].

Re-immobilization should be considered when the original immobilization device no longer reproduces the planned geometry. This may occur because of weight loss, tumor regression, edema reduction, patient discomfort, or progressive loosening of the mask. In such cases, repeated daily corrections may temporarily hide the underlying problem; however, they do not restore the relationship among the patient, mask, and planned dose distribution. A new mask, additional support, or repeat simulation may be required when reproducibility cannot be achieved by repositioning alone [4,16].

Adaptive replanning becomes relevant when the issue is no longer about setup but an anatomical change that alters the delivered dose. Examples include target shrinkage, nodal regression, parotid medial shift, body contour change, or persistent separation between the planning CT and the current treatment anatomy. In these situations, the clinical question is not only whether alignment can be achieved but also whether the original plan remains dosimetrically appropriate. Trigger-based adaptive radiotherapy provides a practical framework for this decision, because it aims to identify patients with large anatomical or positional changes that require repeat imaging, replanning, or both [4,5,62,63,64,65,66].

However, the central issue is not simply which action is selected, but how the need for action is judged. In this context, IGRT should not be regarded merely as an imaging or registration technique. In head and neck radiotherapy, IGRT should also be understood as a clinical judgment process performed at the treatment unit, in which radiation therapists determine whether the daily patient geometry is acceptable for the specific treatment intent and dose distribution. Perfect reproduction of all anatomical regions is desirable but not always achievable, because the target is a living human body with region-dependent motion, deformation, and anatomical change. Therefore, the key technical skill is not simply to minimize the global registration error, but to understand which anatomical region must be prioritized for each patient.

For example, in nasopharyngeal cancer with skull-base or clival invasion, the high-dose CTV may be located immediately adjacent to the brainstem or intracranial structures. In such cases, the treatment plan often relies on a steep dose gradient to maintain target coverage while respecting the brainstem dose constraint. The radiation therapist performing daily IGRT must therefore understand that even a small residual mismatch around the skull base may be clinically more important than a comparable mismatch in a lower-neck elective nodal region. In contrast, a small residual mismatch in the lower neck may be managed differently when cranial target coverage and brainstem sparing are the dominant priorities. Thus, action-oriented positioning management requires patient-specific prioritization of image matching based on target extent, organs at risk, dose-gradient location, and treatment intent, rather than uniform alignment of the entire head and neck volume.

Therefore, beyond couch correction, positioning management should be understood as a stepwise and multidisciplinary clinical process. First, the treatment team determines whether the observed discrepancy can be adequately managed using couch-based translational or rotational correction. If the discrepancy reflects altered neck posture, shoulder position, mandibular rotation, or regional anatomical mismatch, repositioning is performed to restore the planned treatment posture. If reproducibility remains poor, re-immobilization or repeat simulation should be considered. If anatomical changes compromise target coverage or organ-at-risk sparing, adaptive replanning should be considered and, when appropriate, implemented. Importantly, radiation therapists must perform this process with an understanding of the treatment intent, target extent, planning margins, dose-gradient location, and the priorities embedded in the treatment plan. When residual mismatch cannot be fully corrected, positioning should be guided by patient-specific priorities regarding which anatomical structures must be matched most strictly and which regions may tolerate limited residual uncertainty. This requires a multidisciplinary understanding of the radiation oncologist’s clinical intent and the planner’s dose-distribution strategy. Thus, action-oriented positioning management is not simply a technical procedure for image matching, but a patient-specific positioning skill that integrates daily imaging, repositioning, dose-distribution awareness, and clinical judgment.

## 8. Clinical Impact of Positioning Quality

The clinical importance of positioning quality in head and neck radiotherapy lies in its effect on the delivered dose distribution. Deviations of only a few millimeters can alter the target coverage and organ-at-risk dose, including parotid sparing. Therefore, positioning quality should be regarded not only as a geometric endpoint but also as a determinant of dosimetric treatment quality [1,2,67].

The dosimetric effect of the positioning uncertainty is not uniform. Depending on the anatomical region involved and the structure of the treatment plan, a mismatch may reduce target coverage and/or increase the organ-at-risk dose. Therefore, regional deviations involving the lower neck, shoulders, mandible, or soft tissues may have different clinical consequences, even when the overall setup appears acceptable [10,57,67].

Adaptive radiotherapy provides indirect evidence suggesting that these changes are clinically significant. Published studies and reviews have shown that adaptive replanning can improve target coverage and reduce the dose to at-risk organs, such as the parotid glands and spinal cord, in selected patients. These findings support the concept that deterioration in positioning reproducibility or anatomical fidelity during treatment may justify intervention when it compromises the planned dose distribution [4,64,68,69,70].

The most relevant downstream clinical endpoints are toxicity and tumor control, which should be distinguished from dosimetric endpoints such as target coverage and organ-at-risk dose. Current evidence is strongest for dosimetric effects, more limited for toxicity reduction, and least mature for improved tumor control. Margin reduction combined with daily CBCT-guided VMAT has been associated with reduced radiation-related toxicity, and adaptive radiotherapy reviews have suggested potential benefits for quality of life. However, evidence for improved tumor control remains less mature and should be interpreted cautiously [64,68,71].

Improving positioning quality also incurs practical costs. Frequent volumetric imaging, repeated verification, and adaptive reviews can increase treatment complexity and reduce throughput. Reported online ART workflows have required approximately 20 min per fraction in a head and neck series and 34.5 ± 11.4 min per adaptive session in a broader kV-CBCT implementation study. Repeated CBCT imaging also adds protocol-dependent imaging dose, with reported head and neck kV-CBCT organ doses of 0.03–3.43 mGy per scan [72,73,74]. CBCT-based dose reconstruction and dose-of-the-day estimation may support adaptive decision-making, but these approaches require additional resources and should be balanced against workflow burden and imaging dose [75,76,77].

## 9. Emerging Technologies and Future Directions

Emerging technologies are shifting positioning management in head and neck radiotherapy from a largely reactive process to a more predictive and decision-support-oriented framework. Recent advances in artificial intelligence (AI), accelerated image reconstruction, synthetic imaging, and automated workflow design suggest that future positioning systems may not only detect mismatches, but also help classify their causes, estimate their dosimetric relevance, and guide the next appropriate action. Some enabling technologies, including online adaptive workflows, synthetic imaging, corrected or high-quality CBCT, and rapid replanning, have already entered clinical use in selected institutions or commercial platforms. In contrast, AI-based mismatch classification and automated action selection remain largely investigational, and their clinical value has not yet been established through robust prospective validation [78,79,80,81].

**Figure 2:**
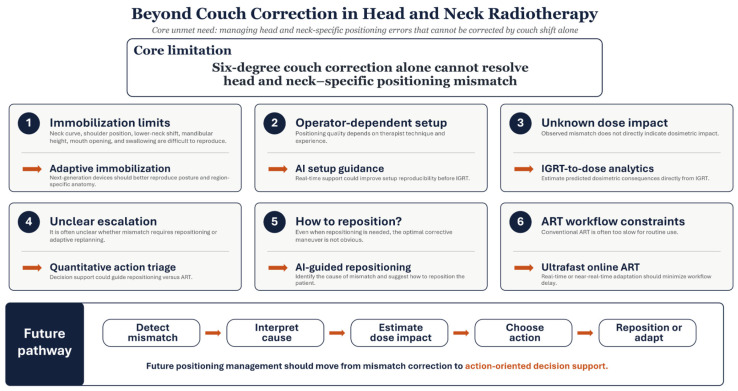
Conceptual framework for action-oriented positioning management beyond couch correction. Six-degree couch correction alone cannot resolve head and neck-specific positioning mismatch. Future technologies should support detection of mismatch, interpretation of its cause, estimation of dose impact, selection of appropriate action, and repositioning or adaptation. This figure was created by the authors and was not reproduced or adapted from previously published material.

Figure 2 summarizes the central unmet need and the major technology-enabled directions for moving beyond couch correction in head and neck radiotherapy. In this framework, emerging tools are not viewed as isolated technical advances; rather, they are organized according to the clinical gap they aim to address: reproducing posture, standardizing setup, estimating dose impact, selecting escalation, guiding repositioning, and enabling rapid adaptation.

One of the most promising developments in this field is AI-based mismatch detection. Machine learning can be used to identify setup-related abnormalities and distinguish them from other causes of treatment deviations. In this context, the significance of AI is not merely that it can automate pattern recognition, but that it may eventually support a more consistent identification of clinically meaningful positioning failures. Related work on in vivo dosimetry tolerance failures also suggests that AI can classify the underlying cause of an observed discrepancy, thereby moving closer to a clinically useful framework of error attribution than to simple error flagging. Nevertheless, most available studies are retrospective and have been evaluated in relatively constrained technical settings rather than in full routine clinical workflows [65,66,78,81,82].

Another step beyond detection is AI-assisted correction suggestion. The central future question is whether a system can move from stating that a mismatch exists to indicating how it should be managed. Current evidence suggests that this transition has started but remains incomplete. Current systems are more likely to generate geometric correction estimates, reconstructed images, or classification outputs than to provide human-readable and clinically actionable repositioning advice, such as whether the patient should be re-setup, whether the chin or neck posture is incorrect, or whether the shoulder position is driving the discrepancy. This constitutes an important knowledge gap. For head and neck radiotherapy, the most clinically valuable future systems may not be those that simply automate correction, but those that can distinguish between rigid error, postural change, immobilization failure, and evolving anatomy, and then recommend appropriate intervention [65,66,78,81]. To make such systems clinically reliable, future studies should use multi-institutional datasets that include variations in immobilization devices, imaging protocols, registration strategies, treatment platforms, and institutional action levels. External validation should evaluate not only geometric prediction accuracy, but also whether AI-assisted recommendations improve action selection, using post-repositioning verification images and subsequent clinical decisions as practical feedback.

Another key frontier is the development of 2D-only or low-information-image-based high-precision positioning. This direction is attractive because it can reduce the imaging burden and improve throughput while preserving geometric accuracy. Although fully mature clinical systems remain limited, recent work on corrected CBCT, synthetic CT generation, and AI-assisted adaptive workflows indicate that clinically useful inference from reduced or degraded imaging information is becoming more feasible. In head and neck radiotherapy, this line of development is particularly relevant because high-frequency volumetric imaging may improve confidence; however, improvement will be at the cost of time, dose, and workflow burden. Future systems capable of extracting actionable geometric or dosimetric information from fewer projections, lower-dose scans, or limited-angle imaging may therefore become highly valuable [65,66,83,84,85,86].

Low-dose and fast CBCT is a highly practical area of development. If image guidance evolves into an adaptive decision platform rather than a simple verification step, repeated volumetric imaging will become more efficient and scalable. Recent studies on CBCT correction algorithms, synthetic CT generation from CBCT, and online adaptive radiotherapy workflows suggest that image quality limitations can increasingly be mitigated computationally. This raises the possibility that lower-dose or faster acquisitions may become sufficient not only for setup verification, but also for dose monitoring and adaptive responses. However, this potential depends on the reliability of the correction algorithms under real-world variations, and is essential to validate whether such reconstructed or enhanced images are robust enough for clinical decision-making across different scanners, institutions, and patient anatomies [83,84,85].

Beyond image reconstruction and enhancement, other emerging technologies may improve the earlier phases of treatment preparation, including initial setup support. Although the head- and neck-specific literature remains less mature in this area than in segmentation or adaptive planning, AI-driven and interactive workflows are increasingly entering the radiotherapy pipeline. These developments suggest that future positioning systems may extend upstream, standardizing the initial posture, reducing operator dependence, and identifying problematic setup conditions before treatment imaging is acquired. Such approaches could be especially valuable at a disease site where reproducibility depends not only on image matching but also on subtle, difficult-to-standardize features, such as neck flexion, mandibular position, shoulder posture, tongue position, swallowing-related motion, and patient cooperation. Since swallowing and tongue motion can produce transient intrafraction displacement even after an apparently acceptable setup and verification, future positioning support may need to incorporate not only static alignment but also motion-aware detection, prediction, or monitoring strategies [17,18,75,86,87,88,89].

However, there are substantial barriers to clinical implementation. Reviews of AI in head and neck oncology have consistently identified limited dataset size, single-institution development, class imbalance, domain shift, insufficient external validation, and poor generalizability as major limitations. These concerns are particularly relevant in positioning-related applications because anatomical heterogeneity, immobilization practices, scanner differences, and patient posture variability may influence model performance. Moreover, AI systems trained on relatively controlled datasets may perform unpredictably when confronted with outlier situations that matter the most in clinical positioning, such as unusual shoulder deformation, mask looseness, substantial weight loss, or atypical neck tilt. Recent evidence also indicates that deep learning performance in related head and neck imaging tasks can be degraded by positional variations such as neck tilt, underscoring the importance of data diversity and anatomical realism in model development [79,80].

Ethical and governance issues should also be considered. A positioning support system that recommends an intervention, alters the clinical workflow, or implicitly prioritizes one registration strategy over another is not simply a technical tool; it becomes a part of the clinical decision-making process. Explainability, accountability, bias control, and prospective oversight are essential. Data sharing and federated learning strategies may help overcome the limitations of small institutional datasets; however, they also introduce challenges in standardization, privacy, and governance. Thus, the development of AI-based positioning support will require not only technical progress but also careful attention to validation design, regulatory expectations, and ethical deployment [76,78].

What matters most is not simply building smarter detection tools but ensuring that these systems can translate observed changes into reliable, clinically actionable guidance. In this field, the most valuable emerging technologies are likely to help the treatment team decide when to reposition and adapt, and when the original plan will no longer be sufficient.

## 10. Conclusions

Positioning in head and neck radiotherapy has evolved substantially with advances in immobilization, image guidance, rotational correction, adaptive workflows, and emerging AI-enabled technologies. Nevertheless, positioning uncertainty remains clinically meaningful because a proportion of relevant mismatches cannot be fully resolved by six-degree-of-freedom couch correction alone. These mismatches may reflect non-rigid posture differences, region-specific deformation, immobilization failure, functional motion, or progressive anatomical change.

Accordingly, the current challenge is no longer limited to detecting setup errors. The more important question is whether the observed mismatch should be managed by online correction, repeat setup, modification of immobilization, repeat simulation, adaptive replanning, or continued treatment with careful monitoring. Although image guidance has become increasingly sophisticated, standardized and actionable support for these decisions remains limited. In this setting, beyond-couch positioning management should be understood as a patient-specific judgment process rather than a purely technical registration task. Radiation therapists must interpret daily images in relation to the planned dose distribution, treatment intent, target extent, organs at risk, planning margins, and dose-gradient location, and determine which anatomical structures require the strictest alignment when perfect anatomical reproduction is not achievable. Thus, action-oriented positioning management is not simply a technical procedure for image matching, but a patient-specific positioning skill that integrates daily imaging, repositioning, dose-distribution awareness, multidisciplinary understanding, and clinical judgment.

Future progress should focus on closing the gap between detection and action. Promising directions include AI-based standardized mismatch detection, AI-assisted repositioning support, dose-impact estimation from IGRT, and workflow-integrated decision systems that distinguish rigid setup errors from posture-related changes, immobilization failures, and evolving anatomy. In parallel, faster CBCT acquisition, automated contouring, rapid replanning, and ultrafast online adaptive radiotherapy may reduce the workflow barriers that currently limit adaptive response. In this context, the most valuable innovation may not simply automate positioning, but help the treatment team choose the next clinically appropriate action.

## Figures and Tables

**Table 1:** Representative regional and functional sources of positioning uncertainty in head and neck radiotherapy.

Study	n	Site	Measurement	Key Quantitative Finding
Motegi et al., 2014 [12]	67	Lower neck	C5	1 SD was 1.9 mm (LR), 1.7 mm (CC), and 2.3 mm (AP).
van Kranen et al., 2009 [14]	38	Lower neck	C5–C7	Systematic/random errors were 1.9/1.7 mm (LR), 1.1/1.9 mm (CC), and 2.2/1.6 mm (AP).
Hirotaki et al., 2023 [10]	20	Lower neck/shoulder/mandible	T1, shoulders, lower jawbone	T1 vertical deviation increased from approximately 3.0 to 4.0–4.2 mm; mandibular roll correlated with parotid D50 in both VMAT and IMPT (r > 0.7).
Neubauer et al., 2012 [6]	10	Shoulder	Humeral heads	Displacement: 2–6 mm; CTV D99 reduction: up to 1.01 Gy.
Hamlet et al., 1994 [17]	NR	Larynx/ Swallowing	Larynx	Laryngeal motion during swallowing: ~20 mm superiorly and <10 mm anteriorly; estimated dose impact: ~0.5%.
van Asselen et al., 2003 [18]	10	Larynx/ Swallowing	Epiglottis tip	Swallowing occupied 0.45% of irradiation time; epiglottis motion was within 7.1 mm for 95% of irradiation time.

## Data Availability

No new data were created or analyzed in this study. Data sharing is not applicable to this article.
